# Cæsarian Section: Both Mother and Child Saved

**Published:** 1846-01

**Authors:** 


*Cæsarian Section: both Mother and Child saved.*—At the Medical Society of the Upper Rhine, M. Dittmar, at the request of the President, gave the following verbal account of a case in which he performed this operation with complete success:

"Barbe Gerber, aged 38, living near St. Marie-aux-mines, of apparently a good constitution; is the wife of a poor day labourer, who supports, with difficulty, a numerous family; her parents, as well as her brothers and sisters, four in number, have always enjoyed good health. In six pregnancies, previous to that of which we are speaking, she carried her infants to the full term, but after the fourth, malacostion, accompanied with chronic bronchitis, made its appearance, recurring with increased severity at every succeeding confinement, so that during she sixth she completely lost the use of her lower extremities; notwithstanding, under the use of cod-liver oil, her state improved very much. The consequence of this disease was a remarkable diminution of stature, and a deformity of the pelvis, rendering the sixth accouchement very tedious; it was, however, accomplished without the aid of a physician.

"During the whole period of the seventh pregnancy, the patient found herself very well, with the exception of some little difficulty in walking. On the 1st of August last she had found labour pains, and on the 2nd, at six in the evening, the membranes ruptured, and a left hand presented. M. Dittmar was not sent for until midnight, when the following was what he found; through the belly, which was very prominent, he felt the head of a fœtus above the upper strait of the pelvis, resting on the pubic arch. The vulva was enormously swollen and between the labia appeared the left hand of the child. The '*toucher*' astonished M. Dittmar by informing him of the extreme narrowing of the biischiadic diameter, and of the closing in of the pubic arch, which would barely admit of the passage of two fingers. It was with great difficulty that he was able to satisfy himself as to the position of the child; the occiput rested on the right half of the symphysis pubis, with the forehead turned towards the left sacro-iliac synchondrosis, also resting on the brim of the pelvis. Strong uterine contractions, quickly succeeding each other, only increased the tumour on the head of the child; the head itself remained fixed. M. Dittmar at first thought of breaking up the head, and then extracting it with the cephalotribe, as he had done in a similar case a few weeks before, but being soon convinced, from the state of the pelvis, that even this operation was impracticable, he had a consultation on the case with M. Wolf.

"The first thing to be done was to learn accurately the dimensions of the pelvis, and the following are the conclusions at which they arrived: The height of the woman is $1^{m}$, 40 ($4^{f}$, 9·11),* the vertebral column presents no deviation from the normal state, the last false ribs on each side touch the internal margin of the crest of the ilium.

"From one anterior superior iliac spine to the other is but $0^{m}$·27 (10·63 in.)

"From sacrum to symphysis pubis, $0^{m}$·18 (7·08 in.)

"From one trochanter to the other, $0^{m}$·27 (10·63 in.)

"The sub-pubic antero-posterior diameter, $0^{m}$·026 (3·38 in.), from which 0·006 (0·23 in.), must be deducted for the soft parts. The right oblique diameter, approximately estimated from external measurement, is $0^{m}$·06 (2·26 in.) and the left a few millimetres more. The coxysub-pubic diameter, $0^{m}$·053 (1·86 in.), and lastly the biischiadic diameter, $0^{m}$·06 (2·26 in.)

The ilio-pubic arch, in place of being widened, presents a marked convexity inwards, greater at right than at left side; in consequence

* The numbers in parentheses are the French measures reduced to English inches and decimal parts.

of this deformity the symphysis is very prominent, projecting in a beak-like process, and its plane is nearly horizontal, its inferior edge being turned towards the sacro-vertebral angle.

These measurements having convinced both practitioners that it was absolutely impossible for the head to pass down into the pelvis and clear the inferior aperture, they thought of the Cæsarian operation as the only means of safety to both mother and child, and proceeded to practise it at noon on the 3d of August.

The incision was made in the linea alba, and extended four or five centimetres (one and a half to two inches,) beyond the umbilicus; a small omental hernia occurred at the superior extremity of the wound, but was easily reduced. The bladder, rising about six centimetres above the pubis, prevented the incision being extended in that direction, and the uterus had to be swayed forward a little in order to carry the incision sufficiently far upwards. A female infant, at the full term and in perfect health, with the exception of a slight depression of the parietal, produced by pressure against the sacro-vertebral angle, was easily removed through the wound which had been made. The umbilical cord was very short, and so frangible that on extending it a little it ruptured in two places. The uterus contracted strongly after being emptied of its contents, but M. Dittmar, apprehensive that the wound would close so much as to prevent the removal of the placenta, brought it away by introducing his hand, it having some points of adherence. The uterine contraction afterwards relaxing unequally at the two edges of the wound, it remained gaping and bossed at one side; to prevent the intestines insinuating themselves into it, it was closed by a single point of interrupted suture, the integuments were brought together by four points of interrupted suture, and the dressing was completed by slips of adhesive plaster covered by charpie and a bandage.

The reaction following the operation was very slight, the lochia made their appearance on the third day, and soon became purulent, at the same time the breasts enlarged, and gave milk freely. Circumscribed peritonitis appeared several times in the hypogastric region, but was dissipated by leeches, cataplasms, and mercurial frictions, constipation was combated by castor-oil and calomel. The only alarming symptom that presented itself was in the chest; the patient, being affected with mucous catarrh, had the respiration very much impeded by the intestines being pushed up towards the chest, and by the copious mucus choking up the bronchi; this state of things was very much improved on the removal of the bandage. Cicatrization of the wound went on rapidly and was completed on the twenty-fifth day, with the exception of a small fistulous opening, which it took two weeks longer to heal. Six weeks after the operation the patient was able to work.—*Gazette Medicale de Strasbourg.*

